# Casein-Derived Lactotripeptides Reduce Systolic and Diastolic Blood Pressure in a Meta-Analysis of Randomised Clinical Trials

**DOI:** 10.3390/nu7010659

**Published:** 2015-01-20

**Authors:** Ágnes A. Fekete, D. Ian Givens, Julie A. Lovegrove

**Affiliations:** 1 Hugh Sinclair Unit of Human Nutrition and Institute for Cardiovascular and Metabolic Research (ICMR), Department of Food and Nutritional Sciences, University of Reading, Reading RG6 6AP, UK; E-Mail: j.a.lovegrove@reading.ac.uk; 2 Food Production and Quality Research Division, School of Agriculture, Policy and Development, Faculty of Life Sciences, University of Reading, Reading RG6 6AP, UK; E-Mail: d.i.givens@reading.ac.uk

**Keywords:** lactotripeptides, isoleucine-proline-proline (IPP), valine-proline-proline (VPP), blood pressure, meta-analysis

## Abstract

There is an urgent need to treat individuals with high blood pressure (BP) with effective dietary strategies. Previous studies suggest a small, but significant decrease in BP after lactotripeptides (LTP) ingestion, although the data are inconsistent. The study aim was to perform a comprehensive meta-analysis of data from all relevant randomised controlled trials (RCT). Medline, Cochrane library, EMBASE and Web of Science were searched until May 2014. Eligibility criteria were RCT that examined the effects of LTP on BP in adults, with systolic BP (SBP) and diastolic BP (DBP) as outcome measures. Thirty RCT met the inclusion criteria, which resulted in 33 sets of data. The pooled treatment effect for SBP was −2.95 mmHg (95% CI: −4.17, −1.73; *p* < 0.001), and for DBP was −1.51 mmHg (95% CI: −2.21, −0.80; *p* < 0.001). Sub-group analyses revealed that reduction of BP in Japanese studies was significantly greater, compared with European studies (*p* = 0.002 for SBP and *p* < 0.001 for DBP). The 24-h ambulatory BP (AMBP) response to LTP supplementation was statistically non-significant (*p* = 0.101 for SBP and *p* = 0.166 for DBP). Both publication bias and “small-study effect” were identified, which shifted the treatment effect towards less significant SBP and non-significant DBP reduction after LTP consumption. LTP may be effective in BP reduction, especially in Japanese individuals; however sub-group, meta-regression analyses and statistically significant publication biases suggest inconsistencies.

## 1. Introduction

Cardiovascular diseases (CVD) remain the major cause of death worldwide, with a further expected increase by 2030 [1]. A large body of evidence suggests that high BP is one of the most important controllable risk factors of CVD, responsible for 13% of deaths in the world annually, and is a leading global risk for mortality [2]. High BP can be asymptomatic, thus many hypertensive individuals are unaware of its existence. However, it may cause serious damage to organs such as the heart, kidneys or eyes. Therefore, even a small reduction in BP of 2–5 mmHg is clinically significant and can have a major impact on public health [3]. Environmental factors, including diet and lifestyle, have a direct effect on hypertension, with health authorities recommending the adoption of a healthy diet and lifestyle for individuals with raised BP, in addition to healthy people, as a preventive measure against the development of hypertension [4].

In the last two decades both scientific and commercial attention has focused on the beneficial effects of milk proteins and peptides on human health as a potential ingredient for functional foods aimed at controlling elevated BP. The release of antihypertensive milk peptides from the intact protein can be achieved by: fermentation with proteolytic starter cultures; hydrolysis by various enzymes produced by microorganisms or enzymatic hydrolysis in the gastrointestinal digestion [5]. lactotripeptides (LTP)—valine-proline-proline (VPP) and isoleucine-proline-proline (IPP), which can be released from β-casein and κ-casein—are the two most investigated casein-derived peptides in both animal and human studies.

To date, there are four meta-analyses that specifically addressed the effect of LTP on BP; however Xu *et al.* [6] included only nine randomised controlled trials (RCT), while Cicero *et al.* [7] performed a meta-analysis on 18 RCT. The most recent meta-analyses were conducted by Turpeinen *et al.* which included 19 RCT [8], and by Qin *et al.* which analysed 24 studies resulting in 28 trials [9]. Despite the growing number of meta-analyses, to date, most included a restricted number of available published studies, limited sub-group, meta-regression and publication bias analyses. Therefore the aim of this study was to conduct a meta-analysis on all available published RCT, which complied with strict predefined criteria, to provide a more accurate estimate of the true treatment effect of LTP ingestion. Furthermore we aimed to carefully scrutinise the relationship between study-level covariates and effect size as well as sub-group analyses and publication bias. Additionally our goal was to review and critically evaluate the existing meta-analyses, which exclusively focused on the effects of LTP on BP. Thus this more precise, comprehensive and independent analysis will help both researchers, policy makers and the industry to determine whether LTP consumption is an effective strategy for hypertension reduction and prevention.

## 2. Experimental Section

### 2.1. Protocol

Both the inclusion criteria and methods of analysis were specified prior to commencement and the PRISMA [10] guidelines were employed for this review.

### 2.2. Eligibility Criteria for Meta-Analysis

The eligibility criteria for studies to be included in the meta-analysis were: RCT that examined the effects of LTP on BP in men and women aged 18 or above, whose outcome measure was a change in SBP or DBP. The primary outcome measure considered in the meta-analysis was data from office BP measurement, however when these were not reported, data from AMBP or home measurements were used. The secondary outcome measure was AMBP. The intervention products had to contain LTP, were orally administered (at any dose or frequency) and the duration of intervention had to be a minimum of 4 weeks. All studies had to include a control arm.

### 2.3. Information Sources and Search Strategy

The literature search was performed using the following electronic databases: MEDLINE, PUBMED, the Cochrane Library, EMBASE and Web of Science using the following terms: *intervention*, *randomised controlled trials*, *clinical trials*, *high blood pressure*, *hypertension*, anti-hypert*, *milk protein*, *milk peptides*, *casein*, hydrolysate, lactotripeptides*, tripeptide*, VPP, IPP, Ile-Pro-Pro, Val-Pro-Pro, fermented, sour, humans (see Supplementary Information). Furthermore, hand-searching was performed on the reference lists of both eligible studies and review articles. In addition, Google and Google Scholar were searched to confirm that the search was complete. Authors were contacted in cases of incomplete information reported in articles or for reprints of articles when they were not retrievable online. The search period covered studies published in any language until 1 May 2014.

### 2.4. Study Selection, Data Collection Process and Data Items

Titles and abstracts were screened to determine whether they met the inclusion criteria; articles which were not relevant were immediately excluded. Manuscripts were reviewed and the following information was extracted: 1) study design (randomised, parallel, crossover); 2) characteristics of the subjects (BP status, age, gender, BMI, treatment with antihypertensive medication); 3) intervention (type of intervention product, duration, frequency, dose, type of placebo); 4) method of BP measurement (office, AMBP, home); 5) change in SBP and DBP from baseline, and change in BP compared to placebo group. All literature was selected and assessed by AAF, with two further researchers (JAL and DIG) independently reviewing 20% of all papers to validate the procedure. It is common practice that if discrepancies in decisions between the reviewers occur, a fourth investigator is consulted. However in this analysis this was not required as no discrepancies were identified. Data collection and statistical analysis were conducted by AAF under the supervision of a statistician.

### 2.5. Risk of Bias in Individual Studies

Manuscripts were assessed for potential risk of bias according to PRISMA guidelines [10] and the Cochrane Handbook [11]. Studies that met the inclusion criteria were categorised as high, medium or low quality based on appropriate generation of random allocation sequence, concealment of allocation, blinding, incomplete outcome data, selective reporting, and other bias. A trial was considered to be of low quality when more than three unclear risks or two high risks of bias were identified; of medium quality when less or equal to three unclear risks or one high risk were identified; and of high quality when less or equal to one unclear risk was stated. Low risk was considered when the judgement for a criteria was “yes” (*i.e.*, was the allocation sequence adequately generated?); high risk, when the judgement was “no”; and unclear risk, when it was uncertain [11].

### 2.6. Summary Measures and Synthesis of Results

The outcome measure was the difference in means using random effect model. For parallel trials, the treatment effect was calculated by subtracting the change in SBP or DBP from baseline in treatment group from the change in SBP or DBP from baseline in placebo group. For cross-over trials, the given calculated differences in means were used. When more than one dose was used the data from the highest dose group was extracted for the meta-analysis. In order to impute the standard deviation of the treatment effect, a correlation between pre- and post-BP of 0.5 was used [12]. Q statistics was used to test for heterogeneity and *p* < 0.05 was considered statistically significant. Data analysis and synthesis were performed using Comprehensive Meta-analysis version 2.0 [13].

Pre-specified sub-group analyses (with random effects model) on baseline average BP, duration of intervention, type of LTP production, dose of LTP and countries of studies were conducted. For the analysis of study countries, trials were grouped as Japanese and European studies, since a previous meta-analysis suggested that Japanese and European studies showed remarkable BP lowering after LTP supplementation [7]. Differences between sub-groups were assessed manually by significance tests described by Borenstein *et al.* [14]. We performed random effects meta-regression using weights proportional to the inverse variance on dose of LTP, age and BMI of participants. The extent to which the heterogeneity was explained by the covariates was also calculated. Furthermore, publication bias was assessed both visually and formally by evaluating a funnel plot of mean difference for both SBP and DBP and by statistical tests (Begg and Mazumdar rank correlation test (which computes the rank order correlation (Kendall’s tau b) between the treatment effect and the standard error (which is driven primarily by sample size)), Egger’s test of the intercept (using precision (the inverse of the standard error) to predict the standardized effect) and Duval and Tweedie’s trim and fill (determining where the missing studies are likely to fall, adding them to the analysis, and then recalculating the combined effect.)).

## 3. Results

### 3.1. Study Selection

The search of mainstream and other databases as well as the list of references yielded a total of 249 studies (see Supplemental Figure S1.). Reviewing the titles and abstracts resulted in the exclusion of 192 studies, which did not meet the inclusion criteria (conference abstracts were excluded due to the limited reported information on the trials). Therefore full manuscripts of the remaining 58 articles were obtained in order to examine them in more detail. A total of seven [15,16,17,18,19,20,21] studies were excluded as they appeared to use whey protein or associated peptides as an intervention product. The remaining 53 studies used casein or its associated peptides. Of the 52 studies identified, 45 used LTP and five used other peptides such as C12 peptide (Phe-Phe-Val-Ala-Pro-Phe-Pro-Glu-Val-Phe-Gly-Lys) [22,23,24,25], or serine-lysine-valine-tyrosine-proline peptide [26], and two used intact casein [20,21], which were excluded from the analysis.

A total of 30 studies met the selection criteria. The reason for study exclusion included studies which were not randomised (*n* = 3) [27,28,29], trials investigating postprandial responses (*n* = 1) [30], preliminary studies (*n* = 3) (no control group used) [31,32,33], inclusion of plant sterols in the intervention product (*n* = 2) [34,35], duplicate publication (*n* = 1) [36], no measure of BP (*n* = 1) [37], insufficient data (*n* = 2) [38,39], intervention duration was less than four weeks [40,41], and in Japanese and unable to be translated (*n* = 1) [42]. When the nutritional intervention included LTP and LTP with other intervention (e.g., exercise) [43,44] or substances [45,46] (e.g., other LTP or K^+^), only data from LTP supplementation arm and control were used. When different doses of LTP were tested, the result from the highest dose was included [47,48,49,50,51].

### 3.2. Study Characteristics

The 30 studies involving 33 clinical trials were published between 1996 and 2014 (see Table 1.). The majority of studies used a parallel design and double blinding, however five studies used cross-over design [45,46,52,53,54], three trials were single blinded [43,44,48] and one failed to specify blinding [54]. All cross-over studies involved wash-out periods ranging from one to four weeks, apart from one study [45], which did not have a wash-out period, justifying it with the short half-life of LTP, and another study did not mention a wash-out period [54]. The duration of interventions varied between 4–21 weeks and the average duration was 7.8 weeks.

The 30 papers included a total of 2200 (1184 males, 1016 females) participants (who were randomised to treatments), with a mean of 64.7 subjects per trial. The average dose of LTP was 10.5 mg/day (5.2 mg/day of IPP and 5.3 mg/day of VPP). Of the studies included in the meta-analysis, 14 (47%) were performed in Japan [43,44,48,55,56,57,58,59,60,61,62,63,64,65], six (20%) in Finland [50,51,52,66,67,68], four (13%) in the Netherlands [45,49,69,70], three (10%) in Italy [53,54,71], one (3%) in the USA [72], one (3%) in Denmark [47] and one (3%) in Scotland [46]. The baseline SBP of participants ranged between 110–160 mmHg and baseline DBP between 65–95 mmHg. Only 9 studies reported their results by 24-h AMBP, which resulted in 10 sets of data [46,47,50,51,53,54,67,71]. Germino *et al.* failed to provide DBP results [72]. The study of Engberink *et al.* was excluded from this analysis, as they failed to report AMBP results in an appropriate manner to include into our meta-analysis. Two studies included participants who took anti-hypertensive medication, however the dose and type of medication did not change during intervention [55,66].

The intervention products were produced by enzymatic hydrolysis and by fermentation in 15 and 13 trials, respectively, one [69] used three types of LTP products by three different types of production method (fermentation, enzymatic hydrolysis and synthetic); and another three [43,44,72] did not specify the production process of the product. The food matrices which carried LTP were varied: 10 trials involved tablets or capsules [41,43,44,45,48,56,58,61,64,65], nine used milk drinks [47,50,51,52,55,60,66,67,68], six involved fruit and vegetable juices [53,54,62,63,71,72], and five involved yoghurt drinks [47,49,60,69,70]. The primary outcome of the majority of trials was BP change, however the primary outcome of three [63,64,65] studies was the safety of excessive LTP intake; and three had vascular reactivity as primary outcomes [43,44,50].

### 3.3. Summary Results of the Effect of LTP Intervention

**Figure 1:**
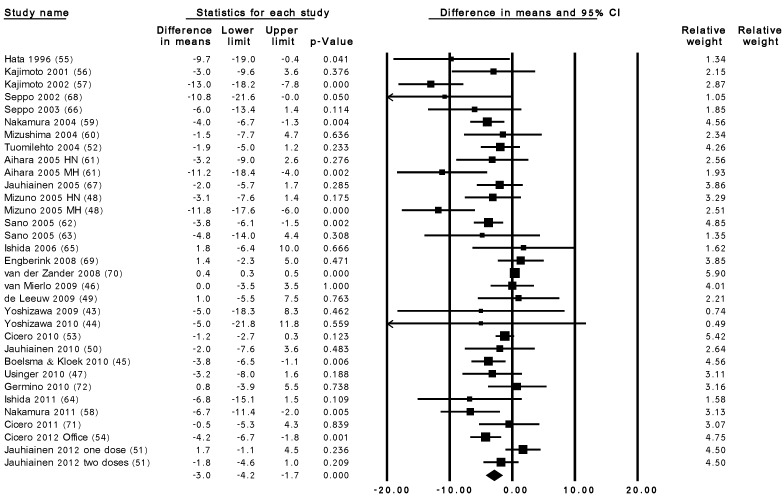
Overall change in SBP (mmHg) after LTP supplementation. HN, high-normotensives; MH, mild-hypertensives; 0.000 refers to p<0.001. Weighted mean difference (95% CI), random effects model. Q = 141.01, df = 32, *p* < 0.001, I^2^ = 77%.

**Figure 2:**
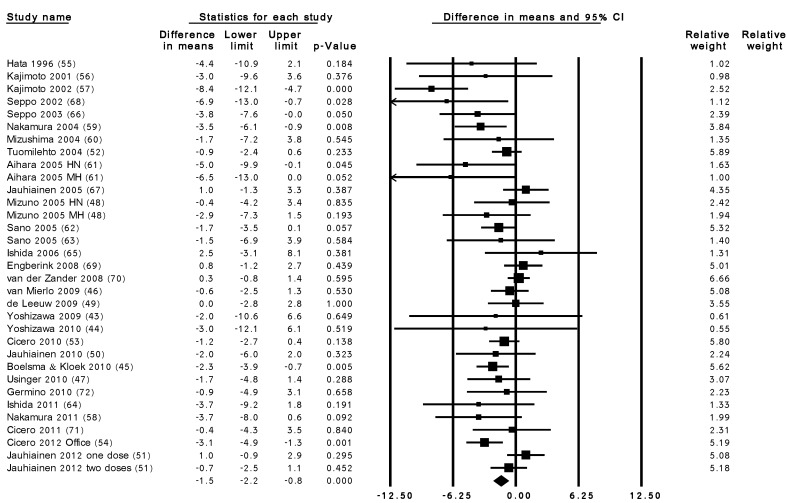
Overall change in DBP (mmHg) after LTP supplementation. HN, high-normotensives; MH, mild-hypertensives; 0.000 refers to p<0.001. Weighted mean difference (95% CI), random effects model. Q = 62.13, df = 32, *p* = 0.001, I^2^ = 49%.

In pooled analysis, LTP supplementation reduced office SBP by 2.95 mmHg (95% CI: −4.17, −1.73; *p* < 0.001) and reduced office DBP by 1.51 mmHg (95% CI: −2.21, −0.80; *p* < 0.001). The pooled effects both for SBP (I^2^ = 77%, Tau^2^ = 6.6, Chi^2^ = 141.0, degree of freedom (df) = 32, *p* < 0.001) and DBP (I^2^ 48%, Tau^2^ = 1.6, Chi^2^ = 62.1, df = 32, *p*-value = 0.001) were heterogeneous (Figure 1 and Figure 2). Using AMBP results, LTP supplementation showed a tendency for reduction in both SBP (−0.94 mmHg, 95% CI: −2.06, 0.18, *p* = 0.101) and DBP (−0.46 mmHg, 95% CI: −1.11, 0.19, *p* = 0.166) although this did not reach statistical significance. The pooled effects for both SBP (I^2^ = 29%, Tau^2^ = 0.9, Chi^2^ = 12.6, df = 9, *p* = 0.182) and DBP (I^2^ = 4%, Tau^2^ = 0.1, Chi^2^ = 8.3, df = 8, *p* = 0.040) appear to be homogenous.

**Table 1:** Trial characteristics of studies included in the meta analysis ^1^.

Reference	Subject Group, *n* (M/F), Average Age and BMI	Study Design and Duration	Country	Treatment	Tripeptide (mg/day)		Baseline		BP Measure-Ment ^3^
					IPP	VPP	SBP (mmHg) ^2^	DBP (mmHg) ^2^	
Hata *et al.* 1996 [55]	hypertensive, 30 (8/22), (26 on BP ↓ medicine), 74.8 years, 20.5 kg/m^2^	R, NR, C, PAL; 8 weeks	Japan	*L. helveticus* and *S. cerevisiae*; 1 × 100 mL milk drink	1.10	1.50	T: 158.5 ± 45.8 C: 150.9 ± 34.2	88.7 ± 38.8 87.0 ± 32.8	Office
Kajimoto *et al.* 2001 [56]	normotensive, 43 (20/23), 29.7 years, 21.5 kg/m^2^	R, D, C, PAL; 2 weeks	Japan	*L. helveticus* CM4; 1 × 6 tablets	4.50	8.10	T: 113.6 ± 12.0 C: 114.1 ± 10.5	67.3 ± 8.7 68.1 ± 7.6	Office
Kajimoto *et al.* 2002 [57]	mild hypertensive, 64 (33/31), 50 years, 25.1 kg/m^2^	R, D, C, PAL; 8 weeks	Japan	*L. helveticus* and *S. cerevisiae*, *L. helveticus* CM4, *L. delbrueckii bulgaricus*, *S. thermophilus* 2 × 150 mL yoghurt drink	1.58	2.24	T: 148 ± 9 C: 148 ± 9	94 ± 7 95 ± 7	Office
Seppo *et al.* 2002 [68]	mild hypertensive, 17 (5/12), 47.5 years, 27.2 kg/m^2^	R, D, C, PAL; 8 weeks	Finland	*L. helveticus* LBK-16 H; 1 × 150 mL milk drink	2.25	3–3.75	T: 148 ± 12.6 C: 148 ± 13.3	94 ± 6.3 93 ± 2.7	Office
Seppo *et al.* 2003 [66]	hypertensive, 39 (19/20), (16 on BP ↓ medicine), 49.4 years, NR	R, D, C, PAL; 21 weeks	Finland	*L. helveticus* LBK-16 H; 1 × 150 mL milk drink	2.25	3.00	T: 152 ± 12.7 C: 149 ± 11.1	96 ± 5.2 95 ± 5.8	Home
Nakamura *et al.* 2004 [59]	high-normotensive, 106 (34/72), 38.5 years, 22.1 kg/m^2^	R, D, C, PAL; 12 weeks	Japan	*L. helveticus* and *S. cerevisiae*, *L. helveticus* CM4, *L. delbrueckii bulgaricus*, *S. thermophilus* 2 × 150 mL yoghurt drink	1.48	2.26	T: 134 ± 5 C: 135 ± 4	79 ± 5 78 ± 5	Office
Mizushima *et al.* 2004 [60]	hypertensive, 46 M, 46.5 years, 25 kg/m^2^	R, D, C, PAL; 4 weeks	Japan	*L. helveticus* and *S. cerevisiae*; 1 × 160 mL milk drink	1.15	1.98	T: 147.6 ± 9.6 C: 145.3 ± 13.0	95.3 ± 9.9 91.5 ± 9.6	Office
Toulomilehto *et al.* 2004 [52]	mild hypertensive, 59 (36/23), 52.8 years, 28.6 kg/m^2^	R, D, C, CO; 13–17 weeks	Finland	*L. helveticus* LBK-16 H; 1 × 150 mL milk drink	2.4–2.7	2.4–2.7	T: 152.7 ± 10.0 C: 156.6 ± 11.4	98.1 ± 7.2 98.1 ± 7.0	Office
Aihara *et al.* 2005 [61]	high-normotensive, 40 (26/14), 51.4 years, 24.2 kg/m^2^	R, D, C, PAL; 4 weeks	Japan	*L. helveticus* CM4; 1 × 6 tablets	4.70	8.30	T: 137.4 ± 5.1 C: 136.8 ± 6.1	85.1 ± 4.9 84.8 ± 11.0	Office
	mild hypertensive, 40 (32/8), 51.7 years, 25 kg/m^2^						T: 148.8 ± 7.2 C: 146.6 ± 8.7	92.6 ± 11.9 92.1 ± 8.7	
Jauhiainen *et al.* 2005 [67]	hypertensive, 108 (69/39), 53 years, 28.5 kg/m^2^	R, D, C, PAL; 10 weeks	Finland	*L. helveticus* LBK-16 H; 2 × 150 mL milk drink	IPP	VPP	SBP (mmHg)^2^	DBP (mmHg)^2^	Office
Mizuno *et al.* 2005 ^4^ [48]	high-normotensive, 24 (6/18), 42.8 years, 25 kg/m^2^	R, S, C, PAL; 6 weeks	Japan	*A. oryzae* hydrolysate; 1 × 2 tablets	1.76	1.86	T: 133.9 ± 6.7 C: 132.8 ± 3.4	81.2 ± 4.7 80.2 ± 2.7	Office
	mild hypertensive, 41 (20/21), 45.9 years, 22.8 kg/m^2^						T: 148.0 ± 6.3 C: 148.4 ± 7.6	88.1 ± 6.2 88.6 ± 3.7	
Sano *et al.* 2005 [62]	hypertensive, 144 (57/87), 50.5 years, 24 kg/m^2^	R, D, C, PAL; 12 weeks	Japan	*A. oryzae* hydrolysate; 1 × 200 mL vegetable-fruit juice	1.60	1.47	T: 138.2 ± 6.5 C: 138.5 ± 6.7	84.4 ± 5.3 85.2 ± 5.7	Office
Sano *et al.* 2005 [63]	normotensive and hypertensive, 43 (21/22), 46.9 years, 23.6 kg/m^2^	R, D, C, PAL; 4 weeks	Japan	*A. oryzae* hydrolysate; 3 × 200 mL vegetable-fruit juice	4.80	4.41	T: 132.3 ± 17.0 C: 133.1 ± 15.3	80.6 ± 9.2 82.3 ± 9.5	Office
Ishida *et al.* 2006 [65]	normotensive and hypertensive, 54 (16/38), 51.9 years, 24.9 kg/m^2^	R, D, C, PAL; 4 weeks	Japan	*A. oryzae* hydrolysate; 1 × 20 tablets	10.10	5.65	T: 131.4 ± 15.2 C: 133.4 ± 15.9	81.9 ± 10.8 83.7 ± 10.9	Office
Engberink *et al.* 2008 [69]	hypertensive, 35 (23/12), 58.8 years, 26.9 kg/m^2^	R, D, C, PAL; 8 weeks	the Netherlands	fermentation; 1 × 200 mL low-fat yoghurt drink	4.20	5.80	142.0 ± 11.3	83.2 ± 8.3	Office
	hypertensive, 32 (22/10), 54.2 years, 26.8 kg/m^2^			enzymatic hydrolysis; 1 × 200 mL low-fat yoghurt drink	5.40	5.00	141.6 ± 13.6	85.5 ± 10.8	Office
	hypertensive, 36 (23/13), 59.5 years, 27.0 kg/m^2^			chemical synthesis; 1 × 200 mL low-fat yoghurt drink	5.20	5.00	142.2 ± 12.6	81.9 ± 10.8	Office
	hypertensive, 32 (20/12), 58.9 years, 26.8 kg/m^2^			placebo; 1 × 200 mL low-fat yoghurt drink	0.00	0.00	140.7 ± 13.0	83.9 ± 7.4	Office
van der Zander *et al.* 2008 [70]	mild hypertensive, 275 (153/122), 60 years, 26.7 kg/m^2^	R, D, C, PAL; 8 weeks	the Netherlands	*A. oryzae* hydrolysate; 1 × 200 mL yoghurt drink	5.78	4.40	150 ± 12.8 150 ± 12.9	85.2 ± 9.2 86.2 ± 9.3	Office
van Mierlo *et al.* 2009 ^5^ [46]	mild hypertensive, 69 (45/24), 61.7 years, 26.9 kg/m^2^	R, D, C, CO; 8 weeks	Scotland	*A. oryzae* hydrolysate; 1 × 200 mL yoghurt drink	5.78	4.40	147.4 ± 9.0	89.0 ± 5.9	Office
de Leeuw *et al.* 2009 ^2^ [49]	mild hypertensive, 41 (21/20), 59.5 years, 25 kg/m^2^	R, D, C, PAL; 8 weeks	the Netherlands	*A. oryzae* hydrolysate; 1 × 200 mL yoghurt drink	4.56	4.47	149 ± 15 148 ± 13	85 ± 8 85 ± 9	Office
Yoshizawa *et al.* 2009 ^6^ [43]	normotensive, 28 F, 58 years, 21.7 kg/m^2^	R, S, C, PAL; 8 weeks	Japan	enzymatic hydrolysis; 1 × 8 tablets	4.30	2.40	116 ± 20.8 110 ± 19.0	68 ± 10.4 66 ± 12.6	Office
Yoshizawa *et al.* 2010 ^6^ [44]	normotensive, 22 F, 57.5 years, 22 kg/m^2^	R, S, C, PAL; 8 weeks	Japan	enzymatic hydrolysis; 1 × 8 capsules	4.30	2.40	112 ± 23.4 104 ± 14.4	76 ± 11.7 72 ± 10.8	Office
Cicero *et al.* 2010 [53]	normo- and high-normotensive, 55 (30/25), 40.3 years, 25 kg/m^2^	R, D, C, CO; 4 weeks	Italy	enzymatic hydrolysis; 2 × 250 mL vegetable and fruit juice	2.00	4.00	126.0 ± 15.5	80.3 ± 8.7	Office
Jauhiainen *et al.* 2010 ^4^ [50]	hypertensive, 89 (54/35), 49 years, 28.1 kg/m^2^	R, D, C, PAL; 12 weeks	Finland	*L. helveticus* LBK-16 H; 2 × 200 mL fermented milk	11.60	13.20	151.3 ± 14.8 154.6 ± 13.9	95.2 ± 12.2 94.2 ± 8.8	Office
Boelsma, Kloek 2010 ^7^ [ 45]	stage 1 hypertensive, 26 (17/9), 59 years, 26.5 kg/m^2^	R, D, C, CO; 4 weeks	the Netherlands	enzymatic hydrolysis; 2 ×1 capsules	15.00	-	148.7 ± 11.5	89.2 ± 10.1	Office
Usinger *et al.* 2010 ^4^ [47]	mild hypertensive, 60 (28/32), 54 years, 26.5 kg/m^2^	R, D, C, PAL; 8 weeks	Denmark	fermentation; 1 × 300 mL fermented milk	2.50	1.10	145 ± 10.9 136 ± 8.8	92.6 ± 6.4 89.4 ± 7.6	Office
Germino *et al.* 2010 [72]	hypertensive, 81 (45/36), 57.1 years, 29.5 kg/m^2^	R, D, C, PAL; 6 weeks	USA	2 × 1 (75 mg) powder dissolved in apple juice	NR	NR	156.6 ± 6 154.6 ± 6	92.5 ± 8.3 91.6 ± 9.6	Office
Ishida *et al.* 2011 [64]	normo- and mild hypertensive, 48 (24/24), 49.3 years, 22.4 kg/m^2^	R, D, C, PAL; 4 weeks	Japan	*A. oryzae* hydrolysate; 1 × 20 tablets	9.60	7.50	130.6 ± 16.3 131.8 ± 14.8	80.4 ± 10.6 81.1 ± 9.6	Office
Nakamura *et al.* 2011 [58]	hypertensive, 70 (47/23), 57.8 years, 23.9 kg/m^2^	R, D, C, PAL; 8 weeks	Japan	*A. oryzae* hydrolysate; 1 × 4 tablets	1.90	1.50	146.8 ± 4.4 146.9 ± 4.3	87.5 ± 7.1 88.0 ± 7.7	Office
Cicero *et al.* 2011 [71]	high-normotensive and mild hypertensive, 50 (29/21), 51.2 years, 26.8 kg/m^2^	R, D, C, PAL; 6 weeks	Italy	enzymatic hydrolysis; 1 × 250 mL fruit juice	1.00	2.00	142.0 ± 10.9 141.2 ± 9.6	86.4 ± 8.2 86.6 ± 7.7	ABPM
Cicero 2012 [54]	high-normotensive and mild hypertensive, 164 (101/63), 43.9 years, 25.7 kg/m^2^	R, D, C, CO; 4 weeks	Italy	enzymatic hydrolysis; 1 × 250 mL fruit juice	1.00	2.00	T: 133.5 ± 12.9 C: 132.7 ± 12.5	83.3 ± 8.8 82.8 ± 8.3	Office
Jauhiainen 2012 [51]	mild hypertensive, 89 (54/35), 49 years, 28.1 kg/m^2^	R, D, C, PAL; 12 weeks	Finland	*L. helveticus* LBK-16 H; 1 × 200 mL fermented milk	2.40	2.60	T: 148 ± 7 C: 147 ± 5	95 ± 5 94 ± 4	ABPM
		12 weeks		2 × 200 mL fermented milk	23.20	26.40	T: 147 ± 8 C: 145 ± 8	94 ± 6 93 ± 6	ABPM

^1^ ABPM, ambulatory blood pressure monitor; C, controlled; CO, cross-over; BP, blood pressure; D, double blind; DB, diastolic blood pressure; NR, not reported; PAL, parallel; P, placebo group; R, randomised; S, single blind; SB, systolic blood pressure; T, treatment group; ↓, lowering; ^2^ mean ± SD; ^3^ Reported method of measurement used in meta-analysis; ^4^ Data from participants assigned to the highest dose; ^5^ Data from participants assigned to LTP only; ^6^ Data from participants assigned to LTP without exercise; ^7^ Data from participants assigned to IPP only and with stage I hypertension.

We conducted sub-group analyses (Table 2.) on the 33 included trials. The effect of baseline SBP and DBP did not influence the effect size significantly; however participants with elevated BP at baseline appeared to have a greater SBP and DBP reduction after LTP supplementation. The length of treatment had no statistically significant impact on the effect size; however trials with equal to or shorter than eight weeks treatment reported a greater decrease in BP. A smaller dose of LTP (≤10 mg/day) had significantly greater BP reduction than larger dose (*p* = 0.027 for SBP, *p* = 0.015 for DBP). The method of LTP production showed no significant effect on BP-lowering. The country in which the study was performed significantly influenced the treatment effect. All trials were separated into two categories: Japanese and European countries (we excluded the American trial, due to the lack of other studies in the USA). Although both sub-groups showed statistically significant reduction in BP, trials from Japan resulted in significantly greater BP-lowering compared to studies from European countries (*p* = 0.002 for SBP and *p* < 0.001 for DBP).

**Table 2:** Sub-group analyses.

Sub-group Title	No. of Trials	Effect Size (mmHg) (95% CI)	
		SBP	DBP
Baseline BP			
above 140/90 mmHg	20/12	−3.00 (−4.71, −1.29)	−1.90 (−3.38, −0.42)
below 140/90 mmHg	13/21	−2.68 (−3.61, −1.74)	−1.38 (−2.13, −0.63)
*p*-value *		0.748	0.541
Duration of intervention			
≤8 weeks	25	−3.35 (−4.91, −1.79)	−1.80 (−2.72, −0.89)
>8 weeks	8	−2.18 (−3.72, −0.64)	−1.00 (−2.10, −0.09)
*p*-value *		0.966	0.921
Dose of LTP			
≤10 mg/day	21	−3.81 (−5.30, −2.32)	−2.02 (−2.87, −1.16)
>10 mg/day	11	−1.32 (−2.94, 0.30)	−0.41 (−1.38, 0.57)
*p*-value *		*0.027*	*0.015*
Production of LTP			
Enzymatic hydrolysis	14	−2.77 (−4.48, −1.05)	−1.25 (−2.03, −0.47)
Fermentation	15	−3.82 (−5.80, −1.84)	−2.38 (−3.77, −1.00)
*p*-value *		0.433	0.164
Country of studies			
Japan	16	−5.54 (−7.43, −3.65)	−3.01 (−4.25, −1.78)
European countries	16	−1.36 (−2.53, −0.20)	−0.83 (−1.57, −0.10)
*p*-value *		*0.002*	*<0.001*
Trial size			
<50 participants	15	−4.93 (−6.78, −3.07)	−2.40 (−3.42, −1.39)
≥50 participants	18	−1.99 (−3.31, −0.66)	−1.11 (−1.96, −0.26)
*p*-value *		*0.011*	0.057

* Significant test to investigate the difference of between sub-groups, *p* < 0.05.

Meta-regression analyses on the BMI of participants suggested a statistically significant negative association between BMI and BP lowering of LTP (slope coefficient: −24.8, *p* < 0.001 for SBP; and slope coefficient: −14.4, *p* = 0.001 for DBP) and no association between BP reduction and age (slope coefficient: −6.3, *p* = 0.392 for SBP; and slope coefficient: −5.1, *p* = 0.134 for DBP). Similarly, no statistically significant association was found between treatment effect and LTP dose (slope coefficient: −3.6, *p* = 0.297 for SBP; and slope coefficient: −2.0, *p* = 0.130 for DBP). Study quality analysis was performed, which showed that after removing the low quality trials (*n* = 14) the treatment effect shifted to −1.58 mmHg (95% CI: −2.80, −0.35; *p* = 0.012) for SBP and to −0.82 mmHg (95% CI: −1.60, −0.04; *p* = 0.039) for DBP.

### 3.4. Risk of Bias within and across Studies

The information regarding sequence generation and allocation concealment was rarely reported in the included RCTs. Only six studies described both sufficiently [53,54,58,69,70,71] and only three trials specified the allocation concealment [45,46,49]. Most of the trials lacked detailed information regarding participant blinding or whether outcome data assessors were blinded. Six studies [43,44,48,56,57,68] failed to clarify withdrawals and another [64] appeared to have reporting bias. All trials, apart from Cicero *et al.* 2010 [53], 2011 [71] and 2012 [54], were supported by industrial sponsors who provided the treatment products, furthermore many authors were employees of the sponsor companies. We identified 13 studies of low, 14 of medium and only three of high quality (see Supplemental Table S1.).

The visual assessment of funnel plots (standard error on y axis and difference in means on x axis) (see Supplemental Figures S2 and S3.) suggested asymmetry for both SBP and DBP. Begg and Mazumdar rank correlation test showed that tau for SBP was 0.05 and *p*-value = 0.664 and for DBP was −0.22, *p*-value = 0.070. Egger’s test reported intercept of −1.60 and *p* < 0.001 for SBP; and −1.23 and *p* = 0.01 for DBP. The trim and fill analysis estimated that nine publications might be missing for SBP (therefore adjusted values were −1.64 mmHg, 95% CI: −2.80, −0.47) and 11 for DBP (adjusted values were −0.63 mmHg, 95% CI: −1.41, 0.14). Since the funnel plot asymmetry might also arise from other possible sources [73], we compared the fixed and random effects estimates of the intervention effects. The pooled effect with fixed effects model showed an increase in SBP (0.34 mmHg, 95% CI: 0.24, 0.44; *p* < 0.001) after LTP supplementation, with a significant reduction in DBP (−1.11 mmHg, 95% CI: −1.55, −0.68; *p* < 0.001). Therefore we investigated the “small-study effect”. The sub-group analysis of samples sizes (categorised as small *n* < 50, other *n* ≥ 50) showed that the pooled effect of small studies was −4.93 mmHg, 95% CI: −6.78, −3.07; *p* < 0.001 for SBP, and −2.40 mmHg, 95% CI: −3.42, −1.39; *p* < 0.001 for DBP. Effect of sample size ≥50 for SBP was −1.99 mmHg (95% CI: −3.31, −0.66; *p* = 0.003), and for DBP was −1.11 mmHg (95% CI: −1.96, −0.26; *p* = 0.011). Statistically significant differences between study sizes were identified for SBP (*p* = 0.011) and a near significant difference for DBP (*p* = 0.057). The meta-regression analysis to examine the relationship between study effect and sample size suggested that there was a significant negative association between sample size and treatment effect for SBP (slope coefficient: −4.5, *p* = 0.05) and tended to be significant for DBP (slope coefficient: −2.3, *p* = 0.083).

## 4. Discussion

The aim of this paper was to determine the association between LTP consumption and BP by performing a meta-analysis of the relevant literature to date. This analysis included 30 RCTs, extending previous meta-analyses on LTP supplementation [6,7,8,9] (for comparison of included studies among meta-analyses, see Supplemental Table S2.). We found that LTP supplementation modestly, but significantly reduced SBP (−2.95 mmHg) and DBP (−1.51 mmHg). The significant reductions in SBP and DBP after consumption of LTP observed in this study support previous data from meta-analyses on LTP [6,7,8,9], however to different extents (Supplemental Figures S4 and S5.).

There was statistically significant heterogeneity of treatment effects across studies, which was higher for SBP. The included studies varied in many aspects such as dose, participant characteristics, duration of intervention and food matrices used, which could have contributed to heterogeneity. The higher heterogeneity of SBP is likely to be due to its variable nature compared to DBP [74]. The meta-analysis on 24-h AMBP did not result in statistically significant reductions in BP, which is in part in line with the study of Qin *et al.* [9]. They reported statistically significant reduction in SBP (−1.30, 95% CI: −2.49, −0.11) and non-significant reduction in DBP (−0.57, 95% CI: −1.49, 0.35).

Within our analysis we observed that the country in which studies were performed had a significant influence on treatment effect. This is in line with the findings of Cicero *et al.* where BP lowering of LTP consumption was more evident in Japanese and Finnish studies [7]. Furthermore, in the meta-analysis of Cicero *et al.* which focused on European subjects only, BP-lowering of LTP ingestion was reported (−1.28 mmHg, 95% CI: −2.09, −0.48 for SBP and −0.59 mmHg, 95% CI: −1.18, −0.01 for DBP) [75]. Our sub-group analyses of European studies showed similar results: −1.36 mmHg (95% CI: −2.53, −0.20) for SBP and −0.83 mmHg (95% CI: −1.57, −0.10) for DBP. It is known that there are ethnic differences in response to cardiovascular drugs, specifically BP-lowering drugs [76], which might be due to genetic polymorphism or environmental factors such as diet. Another possible explanation for the differences in the response in the Japanese studies, compared with European studies, may be the participants’ habitual milk and dairy consumption. The Japanese diets typically contain low quantities of milk and dairy products, which may impact on the BP responses to inclusion of LTP in their diet [77]. In 2009 Japanese fluid milk consumption was 32.2 kg/capita/year, whereas Europe consumed 64.5 kg/capita/year [78]. However it is of note that there was a lack of information on the dietary intake (milk, dairy products, fermented food or salt intake) of the participants in the studies included in this analysis. Lack of dietary data is a substantial limitation as this information would provide valuable data on the possible impact of habitual dietary intake and responsiveness to LTP supplementation. Furthermore, a recent study of Siltari *et al.* [79] found that the peptides can have four different spatial conformations (*cis*/*trans*) owing to the fermentation process (bacteria) or synthesis. This may have important impact on the biological activity of the products produced in different countries, which may explain the different effects of LTP consumption in different countries. Likewise, it is important to note that different forms of LTP have been tested in the studies included in the meta-analysis, which may have also influenced the high variability observed.

We found a significant negative association between BMI and BP lowering effects of LTP (*p* < 0.001 for SBP and *p* = 0.001 for DBP). Although BMI and BP are highly positively correlated [80], our results are contradictory to this dogma. The BMI of Japanese participants were lower (average BMI was 23 kg/m^2^) than the Caucasians (average BMI was 27 kg/m^2^), yet LTP resulted in significant BP lowering in Japanese subjects. It may be that the smaller body weight of Japanese subjects could also influence the effective concentration of the peptides in the body. Nevertheless this paradoxical finding could simply reflect the greater BP reduction reported in the Japanese studies compared with trials from other countries and may support the identified publication bias. However it is important to note that meta-regression analysis has the same disadvantage as epidemiological studies: bias by confounding. It is a recognised “pitfall” in meta-analyses when the predictor is a subject-level characteristic that has been averaged over study subjects [81,82].

We found that low LTP doses are common in the Japanese studies with an average dose of 7.57 mg/day, compared with 11.68 mg/day for European studies. The negative relationship between dose and effect may reflect the reported greater BP reduction in this population. To establish whether there is a true negative relationship between BMI, LTP dose and hypertensive effects of LTP, a dose-dependent study in the same population with varying BMI is required. This will help to reduce population bias and other potential confounding factors between different population groups.

Whilst considering the risk of bias of the included studies, it was found that trial methodological quality was better in the European studies which involved large numbers of participants [46,69,70], yet these studies showed no significant effects of LTP on BP. Thus, it is likely that the smaller studies made the largest contribution to the observed significant treatment effect from the meta-analysis. This is a recognised phenomenon known as the “small-study effect”, which could have impacted significantly on the differential results from the different groups [73]. Our investigation suggested a modest “small-study effect”, which was more evident in DBP. However when the lower quality trials were removed, and the meta-analysis was repeated, a statistically significant reduction in BP remained. This is in part consistent with our trim and fill analysis, which enables us to estimate how the treatment effect would shift if all studies were available and included in the meta-analysis (assuming no publication bias). Taken together, we concluded that it is likely that some bias influenced the treatment effect, and if so, this might shift the pooled effect of LTP supplementation towards a less significant reduction in SBP and non-significant reduction in DBP.

A key conclusion of this meta-analysis is that inclusion of LTP in the diet has significant hypotensive effects. Although the effect is usually less than many antihypertensive drugs (all drugs total −14.5 mmHg in SBP and −10.7 mmHg in DBP [83]), LTP represents a potentially important prophylactic strategy for reducing the risk of hypertension through the lifespan, which could reduce the need for antihypertensive medication later in life. Moreover, the use of such drugs has been associated with adverse effects such as dizziness, headache and swelling and even hypotension in normotensive individuals [84] which have not been reported for LTP use.

## 5. Strengths of the Meta-Analysis

The present study has a number of strengths and presents the most comprehensive treatment effect of LTP. This study used the most thorough and precisely defined inclusion criteria and followed the PRISMA guideline. Similarly, the literature search was rigorous and robust. This meta-analysis included all available RCT to date that met our robust criteria for inclusion, resulting in 30 trials with 33 sets of data. The high number of RCT allowed the most comprehensive subgroup, meta-regression analyses and publication bias, which provided possible reasons for the observed heterogeneity in treatment effects. The LTP treatment effects were analysed using both office and AMBP, which is also unique.

## 6. Limitation of the Meta-Analysis

The main limitation was the lack of available data in the published manuscripts, which was due in a large part from poor reporting (e.g., lack of detail on statistical analysis, random sequence generation, allocation concealments and blinding of outcome assessments as well as the presentation of the results), particularly in the Japanese papers. Poor reporting style does not necessary reflect poor methodological research. However it might substantially affect the outcome of meta-analyses, and thus the future implications for practice. Therefore it is of paramount importance that manuscripts are written in an appropriate scientific style and format. Furthermore, the format in which the data were presented in many of the papers made data extraction difficult and may have influenced the extracted result. Contacting authors was not successful in every case, which could have reduced the possible errors of the analyses.

## 7. Conclusions

Supplementation of pre- or hypertensive groups with LTP for longer than four weeks resulted in a small, but significant reduction in SBP and DBP. However, when the potential biases were considered, the overall effect of LTP ingestion on BP appeared lower. Further studies are warranted to investigate the possible synergistic effects of LTP with some antihypertensive drugs in well-powered RTC. It is recommended that well designed trials with good reporting style and comprehensive information including ethnicity of studied groups and dietary intake of participants (e.g., milk and dairy consumption, salt intake) at baseline and during the intervention should be conducted. This will lead to a better understanding of the impact of LTP ingestion in BP therapy.

## Supplementary Files

Supplementary File 1
