# Supplementary material for: Casein-Derived Lactotripeptides Reduce Systolic and Diastolic Blood Pressure in a Meta-Analysis of Randomised Clinical Trials
Source: Nutrients. 2015 Jan 20;7(1):659–81. doi: 10.3390/nu7010659 (PMC4303860; doi:10.3390/nu7010659)
Supplement: Supplementary File 1 [file nutrients-07-00659-s001.docx]

**Supplementary Information**

Search term utilised on 01 May 2014

(milk protein OR milk peptides OR casein OR hydrolysate OR lactotripeptide* OR tripeptide* OR IPP OR VPP OR Ile-Pro-Pro OR Val-Pro-Pro OR fermented OR sour) and ((blood pressure OR hypertension OR anti-hypert*)) AND (Humans[Mesh] AND ( Randomized Controlled Trial[ptyp] OR Clinical Trial[ptyp] OR Clinical Trial, Phase I[ptyp] OR Clinical Trial, Phase II[ptyp] OR Clinical Trial, Phase III[ptyp] OR Clinical Trial, Phase IV[ptyp] OR Controlled Clinical Trial[ptyp])).

**Excluded in abstract review *n* = 191**

Irrelevant studies (*n* = 158)

Milk proteins were used as placebo (*n* = 17)

No specification of milk protein used (*n* = 13) Conference abstracts (*n* = 3)

**Search results combined (*n* = 249)**

**RCTs retrieved for more detailed evaluation**

**(*n*=58)**

**Literature search**

Mainstream databases, other databases and reference lists

RRC

**RCTs on casein and its associated peptides**

***N* = 53**

**Excluded RCTs: whey protein and its associated peptides**

***N* = 5**

RRC

**Excluded from meta-analysis: *n* = 23**

Intervention with intact casein or not IPP & VPP peptides (*n* = 7)

Not randomized trials (*n* = 3)

Acute (*n* = 1)

Preliminary study (*n* = 3)

Additional bioactive component present in treatment product (*n* = 2)

Double publication (*n* = 1)

No BP measurement (*n* = 1)

Insufficient data (*n* = 2)

Japanese (*n* = 1)

Intervention length < 4 weeks (*n* = 2)

**Meta-analysis: RCTs on LTPs**

***N* = 30**

**Figure S1.** Flow diagram of the RCT selection process.

**Figure S2.** Missing studies (full circles) adjusted funnel plot for SBP. Observed values of overall effect: −2.95 mmHg (95% CI: −4.17; −1.73), Adjusted values of overall effect:
−1.64 mmHg (95% CI: −2.80; −0.47). Weighted mean difference.

**Figure S3.** Missing studies (full circles) adjusted funnel plot for DBP. Observed values of overall effect: −1.51 mmHg (95% CI: −2.21; −0.81), Adjusted values of overall effect:
−0.63 mmHg (95% CI: −1.41; 0.14). Weighted mean difference.

**Figure S4.** Comparison of treatment effects across meta-analyses (SBP).

**Figure S5.** Comparison of treatment effects across meta-analyses (DBP).

**Table S1.** Risk of bias within trials included in meta-analysis.

| **Reference** | **Random Sequence Generation** | **Allocation Concealment** | **Blinding of Participants** | **Blinding of Investigators** | **Blinding of Outcome Assessment** | **Incomplete Outcome Data** | **Selective Reporting** | **Other Bias** | **Quality of Studies** |
| --- | --- | --- | --- | --- | --- | --- | --- | --- | --- |
| Hata 1996 [1] | Unclear risk | Unclear risk | Unclear risk | Unclear risk | Unclear risk | Low risk | Low risk | Unclear risk | Low |
| Kajimoto 2001 [2] | Unclear risk | Unclear risk | Low risk | Low risk | Unclear risk | High risk | Low risk | Unclear risk | Low |
| Kajimoto 2002 [3] | Unclear risk | Unclear risk | Low risk | Low risk | Unclear risk | High risk | Low risk | Unclear risk | Low |
| Seppo 2002 [4] | Unclear risk | Unclear risk | Low risk | Low risk | Unclear risk | High risk | Low risk | Low risk | Low |
| Seppo 2003 [5] | Unclear risk | Unclear risk | Low risk | Low risk | Unclear risk | Low risk | Low risk | Low risk | Medium |
| Nakamura 2004 [6] | Unclear risk | Unclear risk | Low risk | Low risk | Unclear risk | Unclear risk | Low risk | Unclear risk | Low |
| Mizushima 2004 [7] | Unclear risk | Unclear risk | Low risk | Low risk | Unclear risk | Low risk | Low risk | Low risk | Medium |
| Toulomilehto 2004 [8] | Unclear risk | Unclear risk | Low risk | Low risk | Unclear risk | Low risk | Low risk | High risk | Low |
| Aihara 2005 [9] | Unclear risk | Unclear risk | Low risk | Low risk | Unclear risk | Low risk | Low risk | Low risk | Medium |
| Jauhiainen 2005 [10] | Unclear risk | Unclear risk | Low risk | Low risk | Unclear risk | Low risk | Low risk | Low risk | Medium |
| Mizuno 2005 [11] | Unclear risk | Unclear risk | Low risk | High risk | Unclear risk | High risk | Low risk | Low risk | Low |
| Sano 2005 [12] | Unclear risk | Unclear risk | Low risk | Low risk | Unclear risk | Unclear risk | Low risk | Low risk | Low |
| Sano 2005 [13] | Unclear risk | Unclear risk | Low risk | Low risk | Unclear risk | Low risk | Low risk | Low risk | Medium |
| Ishida 2006 [14] | Unclear risk | Unclear risk | Low risk | Low risk | Unclear risk | Low risk | Low risk | Low risk | Medium |
| Engberink 2008 [15] | Low risk | Low risk | Low risk | Low risk | Low risk | Low risk | Low risk | Low risk | High |
| van der Zander 2008 [16] | Unclear risk | Unclear risk | Low risk | Low risk | Unclear risk | Low risk | Low risk | Low risk | Medium |
| van Mierlo 2009 [17] | Low risk | Unclear risk | Low risk | Low risk | Low risk | Low risk | Low risk | Low risk | High |
| de Leeuw 2009 [18] | Low risk | Unclear risk | Low risk | Low risk | Unclear risk | Low risk | Low risk | Low risk | Medium |
| Yoshizawa 2009 [19] | Unclear risk | Unclear risk | Low risk | Unclear risk | Unclear risk | High risk | Low risk | Unclear risk | Low |
| Yoshizawa 2010 [20] | Unclear risk | Unclear risk | Low risk | Unclear risk | Unclear risk | High risk | Low risk | Unclear risk | Low |
| Cicero 2010 [21] | Low risk | Low risk | Low risk | Low risk | Unclear risk | Unclear risk | Low risk | Unclear risk | Medium |
| Jauhiainen 2010 [22] | Unclear risk | Unclear risk | Low risk | Low risk | Unclear risk | Low risk | Low risk | Low risk | Medium |
| Boelsma, Kloek 2010 [23] | Low risk | Unclear risk | Low risk | Low risk | Unclear risk | Unclear risk | Unclear risk | High risk | Low |
| Usinger 2010 [24] | Low risk | Unclear risk | Low risk | Low risk | Unclear risk | Low risk | Low risk | Low risk | Medium |

**Table S1.** *Cont.*

| Germino 2010 [25] | Unclear risk | Unclear risk | Low risk | Low risk | Unclear risk | Low risk | Unclear risk | Unclear risk | Low |
| --- | --- | --- | --- | --- | --- | --- | --- | --- | --- |
| Ishida 2011 [26] | Unclear risk | Unclear risk | Low risk | Low risk | Unclear risk | Low risk | High risk | Low risk | Low |
| Nakamura 2011 [27] | Low risk | Low risk | Low risk | Low risk | Unclear risk | Low risk | Low risk | Low risk | High |
| Cicero 2011 [28] | Low risk | Low risk | Low risk | Low risk | Unclear risk | Low risk | Unclear risk | Unclear risk | Medium |
| Cicero 2012 [29] | Low risk | Low risk | Low risk | Low risk | High risk | Low risk | Low risk | Unclear risk | Medium |
| Jauhiainen 2012 [30] | Unclear risk | Low risk | Low risk | Low risk | Unclear risk | Low risk | Low risk | Low risk | Medium |

**Table S2.** Comparison of included studies among meta-analyses.

| **Xu 2008 [31]** | **Cicero 2011 [32]** | **Turpeinen 2013 [33]** | **Qin 2013 [34]** | **Fekete 2015** |
| --- | --- | --- | --- | --- |
| Hata 1996 [1] | Hata 1996 [1] | Hata 1996 [1] | Hata 1996 [1] | Hata 1996 [1] |
| Seppo 2002 [4] | Seppo 2002 [4] | Seppo 2002 [4] | Seppo 2002 [4] | Seppo 2002 [4] |
| Seppo 2003[5] | Seppo 2003 [5] | Seppo 2003 [5] | Seppo 2003 [5] | Seppo 2003 [5] |
| Mizushima 2004 [7] | Mizushima 2004 [7] | Mizushima 2004 [7] | Mizushima 2004 [7] | Mizushima 2004 [7] |
| Tuomilehto 2004 [8] | Tuomilehto 2004 [8] | Tuomilehto 2004 [8] | Tuomilehto 2004 [8] | Tuomilehto 2004 [8] |
| Aihara 2005 [9] | Aihara 2005 [9] | Mizuno 2005 [11] | Aihara 2005 [9] | Aihara 2005 [9] |
| Jauhiainen 2005 [10] | Jauhiainen 2005 [10] | Sano 2005 [12] | Jauhiainen 2005 [10] | Jauhiainen 2005 [10] |
| Mizuno 2005 [11] | Mizuno 2005 [11] | Engberink 2008 [15] | Mizuno 2005 [11] | Mizuno 2005 [11] |
| Sano 2005 [12] | Sano 2005 [12] | van der Zander 2008 [16] | Sano 2005 [12] | Sano 2005 [12] |
|  | Engberink 2008 [15] | van Mierlo 2009 [17] | Engberink 2008 [15] | Engberink 2008 [15] |
|  | van der Zander 2008 [16] | Kajimoto 2002 [3] | van der Zander 2008 [16] | van der Zander 2008 [16] |
|  | van Mierlo 2009 [17] | de Leeuw 2009 [18] | van Mierlo 2009 [17] | van Mierlo 2009 [17] |
|  | Cicero 2010 [21] | Nakamura 2004 [6] | Cicero 2010 [21] | Cicero 2010 [21] |
|  | de Leeuw 2009 [18] | Jauhiainen 2010 [22] | de Leeuw 2009 [18] | Kajimoto 2001 [2] |
|  | Yoshizawa 2009 [19] | Hirata 2002* [33] | Yoshizawa 2009 [19] | Kajimoto 2002 [3] |

**Table S2.** *Cont.*

|  | Hirata 2002 [35]* | van der Zander 2008 [36]* | Germino 2010 [25] | de Leeuw 2009 [18] |
| --- | --- | --- | --- | --- |
|  | Kajimoto 2001 [37]* | Turpeinen 2009 [38]* | Usinger 2010 [24] | Yoshizawa 2009 [19] |
|  | Kajimoto 2001 [39]* | Kajimoto 2001 [37]* | Cicero 2011 [28] | Yoshizawa 2010 [20] |
|  |  | Kajimoto 2001 [39]* | Jauhiainen 2010 [22] | Germino 2010 [25] |
|  |  |  | Boelsma & Kloek 2010 [23] | Usinger 2010 [24] |
|  |  |  | Ishida 2011 [26] | Nakamura 2004 [6] |
|  |  |  | Nakamura 2011 [27] | Sano 2005 [12] |
|  |  |  | Yamasue 2010 [40]* | Ishida 2006 [14] |
|  |  |  | van der Zander [36]* | Cicero 2011 [28] |
|  |  |  |  | Jauhiainen 2010 [22] |
|  |  |  |  | Boelsma & Kloek 2010 [23] |
|  |  |  |  | Ishida 2011 [26] |
|  |  |  |  | Nakamura 2011 [27] |
|  |  |  |  | Cicero 2012 [29] |
|  |  |  |  | Jauhiainen 2012 [30] |

* Trials are not included in this meta-analysis.

**References**

1. Hata, Y.; Yamamoto, M.; Ohni, M.; Nakajima, K.; Nakamura, Y.; Takano, T. A placebo-controlled study of the effect of sour milk on blood pressure in hypertensive subjects. *Am. J. Clin. Nutr*. **1996**, *64*, 767–771.
2. Kajimoto, O.; Aihara, K.; Hirata, H.; Takahashi, R.; Nakamura, Y. Safety evaluation of the tablet containing “Lactotripeptides (VPP, IPP)” on healthy volunteers. *J. Nutr. Food* **2001**, *4*, 37–46.
3. Kajimoto, O.; Kurosaki, T.; Mizutani, J.; Ikeda, N.; Kaneko, K.; Aihara, K.; Yabune, M.; Nakamura, Y. Antihypertensive effects of liquid yogurts containing “lactotripeptides (VPP, IPP)” in mild hypertensive subjects. *J. Nutr. Food* **2002**, *5*, 55–66.
4. Seppo, L.; Kerojoki, O.; Suomalainen, T.; Korpela, R. The effect of a *Lactobacillus helveticus*
   LBK-16 H fermented milk on hypertension—A pilot study on humans. *Milchwissenschaft* **2002**, *57*, 124–127.
5. Seppo, L.; Jauhiainen, T.; Poussa, T.; Korpela, R. A fermented milk high in bioactive peptides has a blood pressure-lowering effect in hypertensive subjects. *Am. J. Clin. Nutr.* **2003**, *77*, 326–330.
6. Nakamura, Y.; Kajimoto, O.; Kaneko, K.; Aihara, K.; Mizutani, J.; Ikeda, N.; Nishimura, A.; Kajimoto, Y. Effects of the liquid yoghurts containing “lactotripeptide (VPP, IPP)” on
   high-normal blood pressure. *J. Nutr. Food* **2004**, *7*, 123–137.
7. Mizushima, S.; Ohshige, K.; Watanabe, J.; Kimura, M.; Kadowaki, T.; Nakamura, Y.; Tochikubo, O.; Ueshima, H. Randomised controlled trial of sour milk on blood pressure in borderline hypertensive men. *Am. J. Hypertens*. **2004**, *17*, 701–706.
8. Tuomilehto, J.; Lindstrom, J.; Hyyrynen, J.; Korpela, R.; Karhunen, M.L.; Mikkola, L.; Jauhiainen, T.; Seppo, L.; Nissinen, A. Effect of ingesting sour milk fermented using Lactobacillus helveticus bacteria producing tripeptides on blood pressure in subjects with mild hypertension. *J. Hum. Hypertens*. **2004**, *18*, 795–802.
9. Aihara, K.; Kajimoto, O.; Hirata, H.; Takahashi, R.; Nakamura, Y. Effect of powdered fermented milk with Lactobacillus helveticus on subjects with high-normal blood pressure or mild hypertension. *J. Am. Coll. Nutr*. **2005**, *24*, 257–265.
10. Jauhiainen, T.; Vapaatalo, H.; Poussa, T.; Kyronpalo, S.; Rasmussen, M.; Korpela, R. *Lactobacillus helveticus* fermented milk lowers blood pressure in hypertensive subjects in 24-h ambulatory blood pressure measurement. *Am. J. Hypertens.* **2005**, *18*, 1600–1605.
11. Mizuno, S.; Matsuura, K.; Gotou, T.; Nishimura, S.; Kajimoto, O.; Yabune, M.; Kajimoto, Y.; Yamamoto, N. Antihypertensive effect of casein hydrolysate in a placebo-controlled study in subjects with high-normal blood pressure and mild hypertension. *Br. J. Nutr*. **2005**, *94*, 84–91.
12. Sano, J.; Ohki, K.; Higuchi, T.; Aihara, K.; Mizuno, S.; Kajimoto, O.; Nakagawa, S.; Kajimoto, Y.; Nakamura, Y. Effect of casein hydrolysate, prepared with protease derived from Aspergillus oryzae, on subjects with high-normal blood pressure or mild hypertension. *J. Med. Food* **2005**, *8*, 423–430.
13. Sano, J.; Ohki, K.; Higuchi, T.; Aihara, K.; Mizuno, S.; Kajimoto, O.; Nakagawa, S.; Kajimoto, Y.; Nakamura, Y. Safety evaluation of excessive intake of drink containing “lactotripeptides (VPP, IPP)” in subjects with normal blood pressure to mild hypertension*. J. Nutr. Food* **2005**, *7*, 17–30.
14. Ishida, Y.; Aihara, K.; Sagitani, A.; Kaneko, K.; Mizutani, J.; Nakamura, K.; Shimura, T.; Yagasaki, K.; Nakamura, Y. Safety evaluation of excessive intake of the tablet containing “lactotripeptides (VPP, IPP)” in subject with normal blood pressure to mild hypertension. *J. Pharmacol. Ther*. **2006**, *34*, 1107–1117.
15. Engberink, M.F.; Schouten, E.G.; Kok, F.J.; van Mierlo, L.A.; Brouwer, I.A.; Geleijnse, J.M. Lactotripeptides show no effect on human blood pressure: Results from a double-blind randomised controlled trial. *Hypertension* **2008**, *51*, 399–405.
16. Van der Zander, K.; Bots, M.L.; Bak, A.A.; Koning, M.M.; de Leeuw, P.W. Enzymatically hydrolyzed lactotripeptides do not lower blood pressure in mildly hypertensive subjects. *Am. J. Clin. Nutr*. **2008**, *88*, 1697–1702.
17. Van Mierlo, L.A.; Koning, M.M.; van der Zander, K.; Draijer, R. Lactotripeptides do not lower ambulatory blood pressure in untreated whites: Results from 2 controlled multicenter crossover studies. *Am. J. Clin. Nutr*. **2009**, *89*, 617–623.
18. de Leeuw, P.W.; van der Zander, K.; Kroon, A.A.; Rennenberg, R.M.; Koning, M.M.
    Dose-dependent lowering of blood pressure by dairy peptides in mildly hypertensive subjects. *Blood Press.* **2009**, *18*, 44–50.
19. Yoshizawa, M.; Maeda, S.; Miyaki, A.; Misono, M.; Choi, Y.; Shimojo, N.; Ajisaka, R.; Tanaka, H. Additive beneficial effects of lactotripeptides and aerobic exercise on arterial compliance in postmenopausal women. *Am. J. Physiol. Heart Circ. Physiol*. **2009**, *297*, 1899–1903.
20. Yoshizawa, M.; Maeda, S.; Miyaki, A.; Misono, M.; Choi, Y.; Shimojo, N.; Ajisaka, R.; Tanaka, H. Additive beneficial effects of lactotripeptides intake with regular exercise on endothelium-dependent dilatation in postmenopausal women. *Am. J. Hypertens.* **2010**, *23*, 368–372.
21. Cicero, A.F.; Rosticci, M.; Veronesi, M.; Bacchelli, S.; Strocchi, E.; Melegari, C.; Grandi, E.; Borghi, C. Hemodynamic effects of lactotripeptides from casein hydrolysate in Mediterranean normotensive subjects and patients with high-normal blood pressure: A randomised, double-blind, crossover clinical trial. *J. Med. Food* **2010**, *13*, 1363–1368.
22. Jauhiainen, T.; Ronnback, M.; Vapaatalo, H.; Wuolle, K.; Kautiainen, H.; Groop, P.H.; Korpela, R. Long-term intervention with Lactobacillus helveticus fermented milk reduces augmentation index in hypertensive subjects. *Eur. J. Clin. Nutr*. **2010**, *64*, 424–431.
23. Boelsma, E.; Kloek, J. IPP-rich milk protein hydrolysate lowers blood pressure in subjects with stage 1 hypertension, a randomised controlled trial. *Nutr. J.* **2010**, *9*, doi:10.1186/1475-2891-9-52.
24. Usinger, L.; Jensen, L.T.; Flambard, B.; Linneberg, A.; Ibsen, H. The antihypertensive effect of fermented milk in individuals with prehypertension or borderline hypertension. *J. Hum. Hypertens*. **2010**, *24*, 678–683.
25. Germino, F.W.; Neutel, J.; Nonaka, M.; Hendler, S.S. The impact of lactotripeptides on blood pressure response in stage 1 and stage 2 hypertensives*. J. Clin. Hypertens*. **2010**, *12*, 153–159.
26. Ishida, Y.; Shibata, Y.; Fukuhara, I.; Yano, Y.; Takehara, I.; Kaneko, K. Effect of an excess intake of casein hydrolysate containing Val-Pro-Pro and Ile-Pro-Pro in subjects with normal blood pressure, high-normal blood pressure, or mild hypertension. *Biosci. Biotechnol. Biochem*. **2011**, *75*, 427–433.
27. Nakamura, T.; Mizutani, J.; Ohki, K.; Yamada, K.; Yamamoto, N.; Takeshi, M.; Takazawa, K. Casein hydrolysate containing Val-Pro-Pro and Ile-Pro-Pro improves central blood pressure and arterial stiffness in hypertensive subjects: A randomised, double-blind, placebo-controlled trial. *Atherosclerosis* **2011**, *219*, 298–303.
28. Cicero, A.F.; Rosticci, M.; Gerocarni, B.; Bacchelli, S.; Veronesi, M.; Strocchi, E.; Borghi, C. Lactotripeptides effect on office and 24-h ambulatory blood pressure, blood pressure stress response, pulse wave velocity and cardiac output in patients with high-normal blood pressure or first-degree hypertension: A randomised double-blind clinical trial. *Hypertens. Res*. **2011**, *34*, 1035–1040.
29. Cicero, A.F.; Rosticci, M.; Ferroni, A.; Bacchelli, S.; Veronesi, M.; Strocchi, E.; Borghi, C. Predictors of the short-term effect of isoleucine-proline-proline/valine-proline-proline lactotripeptdies from casein on office and ambulatory blood pressure in subjects with pharmacologically untreated high-normal blood pressure of first-degree hypertension. *Clin. Exp. Hypertens*. **2012**, *34*, 601–605.
30. Jauhiainen, T.; Niittynen, L.; Oresic, M.; Jarvenpaa, S.; Hiltunen, T.P.; Ronnback, M.; Vapaatalo, H.; Korpela, R. Effects of long-term intake of lactotripeptides on cardiovascular risk factors in hypertensive subjects. *Eur. J. Clin. Nutr*. **2012**, *66*, 843–849.
31. Xu, J.-Y.; Qin, L.-Q.; Wang, P.-Y.; Li, W.; Chang, C. Effect of milk tripeptides on blood pressure: A meta-analysis of randomised controlled trials. *Nutrition* **2008**, *24*, 933–940.
32. Cicero, A.; Gerocarni, B.; Laghi, L.; Borghi, C. Blood pressure lowering effect of lactotripeptides assumed as functional foods: A meta-analysis of current available clinical trials. *J. Hum. Hypertens*. **2011**, *25*, 425–436.
33. Turpeinen, A.M.; Jarvenpaa, S.; Kautiainen, H.; Korpela, R.; Vapaatalo, H. Antihypertensive effects of bioactive tripeptides—Arandom effects meta-analysis. *Ann. Med*. **2013**, *45*, 51–56.
34. Qin, L.-Q.; Xu, J.-Y.; Dong, J.-Y.; Zhao, Y.; van Bladeren, P.; Zhang, W. Lactotripeptides intake and blood pressure management: A meta-analysis of randomised controlled trials. *Nutr. Metab. Cardiovas.* **2013**, *23*, 395–402.
35. Hirata, H.; Nakamura, Y.; Yada, H.; Moriguchi, S.; Kajimoto, O.; Takahashi, T. Clinical effect of new sour milk drink on mild or moderate hypertensive subjects. *J. New Rem Clin*. **2002**, *51*,
    61–69.
36. Van der Zander, K.; Jakel, M.; Bianco, V.; Koning, M. Fermented lactotripeptides-containing milk lowers daytime blood pressure in high normal-to-mild hypertensive subjects. *J. Hum. Hypertens*. **2008**, *22*, 804–806.
37. Kajimoto, O.; Aihara, K.; Hirata, H.; Takahashi, R.; Nakamura, Y. Hypotensive effects of the tablets containing “Lactotripeptides (VPP, IPP)”. *J. Nutr. Food* **2001**, *4*, 51–61.
38. Turpeinen, A.; Kumpu, M.; Rönnback, M.; Seppo, L.; Kautiainen, H.; Jauhiainen, T.; Vapaatalo, H.; Korpela, R. Antihypertensive and cholesterol-lowering effects of a spread containing bioactive peptides IPP and VPP and plant sterols. *J. Funct. Food* **2009**, *1*, 260–265.
39. Kajimoto, O.; Nakamura, Y.; Yada, H.; Moriguchi, S.; Hirata, H.; Takahashi, T. Hypotensive effect of sour milk in subjects with mild or moderate hypertension. *J. Jpn. Soc. Nutr. Food Sci.* **2001**, *54*, 347–354.
40. Yamasue, K.; Morikawa, N.; Mizushima, S.; Tochikub, O. The blood pressure lowering effect of lactotripeptides and salt intake in 24-h ambulatory blood pressure measurements. *Clin. Exp. Hypertens*. **2010**, *32*, 214–220.

© 2015 by the authors; licensee MDPI, Basel, Switzerland. This article is an open access article distributed under the terms and conditions of the Creative Commons Attribution license (http://creativecommons.org/licenses/by/4.0/).
